# Tibiotalocalcaneal arthrodesis versus open reduction and internal fixation in treatment of trimalleolar ankle fractures: A propensity-matched TriNetX database study

**DOI:** 10.1016/j.jcot.2026.103511

**Published:** 2026-06-03

**Authors:** Ronak J. Mahatme, Meredith Murphy, Shawn A. Moore, Anish Gangavaram, Caroline Szabo, Abraar Huq, Richard Laughlin

**Affiliations:** Department of Orthopaedic Surgery, University of Cincinnati College of Medicine, Cincinnati, OH, 45267, USA

**Keywords:** Infections, Reoperation, Arthrodesis, Ankle fractures, Wound healing

## Abstract

**Background:**

Trimalleolar ankle fractures are complex injuries associated with high complication rates and poorer outcomes, particularly in elderly and medically comorbid patients. While open reduction and internal fixation (ORIF) remains the standard treatment, alternative strategies such as tibiotalocalcaneal arthrodesis (TTCA) have been increasingly explored for high-risk populations.

**Methods:**

A retrospective cohort study was performed using the TriNetX Research Network. Adult patients undergoing operative treatment for trimalleolar fractures were identified and stratified by procedure (TTCA versus ORIF). Propensity score matching (1:1) was performed to balance demographics and comorbidities, including age, sex, obesity, tobacco use, osteoporosis, diabetes, dementia, and peripheral vascular disease. Outcomes were assessed at 90 days, 1 year, and 2 years. Primary outcomes included secondary surgery (revision procedures, irrigation and debridement, hardware removal, and below-knee amputation). Secondary outcomes included 90-day infection and wound dehiscence. Risk ratios with 95% confidence intervals were calculated, with p < 0.05 considered significant.

**Results:**

After matching, 210 patients were included in each group. At 90 days, infection and wound complication rates were similar between TTCA and ORIF cohorts. TTCA was associated with significantly higher rates of secondary surgery at 1 year (28.1% vs 17.1%; RR 1.64, p = 0.007) and 2 years (31.4% vs 21.0%; RR 1.50, p = 0.015). Irrigation and debridement was also more frequent in the TTCA cohort at 1 year (RR 2.25, p = 0.012) and 2 years (RR 2.23, p = 0.009). In sensitivity analysis of closed fractures, findings remained consistent, with higher secondary surgery rates in the TTCA group.

**Conclusion:**

In this large propensity-matched study, TTCA may be associated with a higher incidence of secondary surgery—particularly irrigation and debridement—compared with ORIF at one and two years postoperatively, while early wound complication rates were similar. These findings may be influenced by residual confounding despite propensity matching.

## 1 Introduction

Trimalleolar ankle fractures represent a complex subset of ankle injuries and are associated with increased morbidity and poorer functional outcomes compared with other fracture patterns.^1^^,^^2^ Open reduction and internal fixation (ORIF) remains the standard operative treatment; however, it carries substantial complication rates, particularly among elderly patients and those with significant medical comorbidities. Reported complication rates in geriatric patients undergoing ORIF for fragility ankle fractures approach 50%.^3^^,^^4^ Risk is further amplified in high-risk populations. Patients with diabetes mellitus experience markedly increased complication rates—including malunion, nonunion, Charcot neuroarthropathy, infection, and amputation—often compounded by associated neuropathy and vasculopathy.^5^^,^^6^ In diabetic cohorts, complication rates following ankle fracture fixation have been reported as high as 47%, while peripheral vascular disease has emerged as an independent risk factor for amputation after surgery.^5^^,^^7^^,^^8^ These concerns have prompted investigation into alternative surgical strategies for medically complex patients.

Tibiotalocalcaneal arthrodesis (TTCA) has emerged as a potential alternative for the management of unstable ankle fractures, particularly in patients at elevated risk for fixation failure. Compared with ORIF, TTCA offers several theoretical advantages, including reduced reliance on osteoporotic bone for fixation stability, which may help mitigate complications in elderly patients.^9^^,^^10^ In addition, TTCA permits immediate or early postoperative weight-bearing, in contrast to the prolonged non-weightbearing typically required after ORIF, and has been associated with shorter hospital stays in geriatric populations.^9^ TTCA can be performed using a range of surgical techniques. These include formal joint preparation with cartilage removal to achieve a true arthrodesis, as well as hindfoot nail placement without joint preparation.^11^^,^^12^ More recently, minimally invasive approaches to joint preparation have also been described.^12^

Despite the proposed advantages of TTCA over ORIF, comparative outcome data between TTCA and ORIF remain limited and conflicting. Balziano et al. and Georgiannos et al. reported lower complication and reoperation rates with TTCA in elderly patients with fragility fractures of the ankle.^3^^,^^9^ Additionally, TTCA has demonstrated favorable limb salvage rates in diabetic populations.^13^ Conversely, Dawar et al. found increased complications and readmissions associated with TTCA compared with ORIF in patients with trimalleolar fractures.^14^ These discordant findings highlight the need for larger comparative analyses.

The purpose of this study was to compare complication rates following TTCA and ORIF in adult patients with trimalleolar fractures using a large, multicenter database. Outcomes were evaluated within open and closed fracture cohorts after propensity score matching for baseline demographic and comorbidity differences. We hypothesized that complication profiles would differ between operative techniques, with TTCA demonstrating lower complication rates in select patient populations.

## 2 Methods

All procedures followed were in accordance with the ethical standards of the responsible committee on human experimentation (institutional and national) and with the Helsinki Declaration of 1975, as revised in 2008 (5). This retrospective cohort study utilized data from the TriNetX Research Network, accessed on March 25, 2026. TriNetX aggregates de-identified electronic medical records from over 150 million patients across 102 healthcare organizations in the United States. The database includes diagnoses, procedures, medications, laboratory values, and genomic information. Use of de-identified patient records exempted this study from institutional review board approval. TriNetX is compliant with HIPAA and certified to the ISO 27001:2013 standard.

Adult patients aged 18 years or older with trimalleolar ankle fractures who had at least 2-years of follow-up were identified, and cohorts were defined according to operative treatment: open reduction and internal fixation (ORIF) versus tibiotalocalcaneal arthrodesis (TTCA). A sensitivity analysis was performed restricted to only closed fractures. Open fractures were not stratified in the sensitivity analysis due to a limited number of events.

To reduce confounding, propensity score matching was performed using a 1:1 greedy nearest-neighbor algorithm without replacement, applying a caliper of 0.1 pooled standard deviations and a tolerance of 0.01 to ensure close covariate balance, with the TTCA group designated as the exposed cohort. Propensity scores were estimated using a multivariable logistic regression, incorporating age at index surgery, sex, body mass index greater than 30, tobacco use, osteoporosis with and without current pathologic fracture, type 2 diabetes mellitus, type 2 diabetes with neurological complications, type 2 diabetes with circulatory complications, dementia, and peripheral vascular disease. Covariate balance before and after matching was assessed using standardized mean differences (SMDs), with values < 0.10 indicating adequate balance. Although propensity score matching was used to reduce measured confounding, residual confounding may persist due to unmeasured variables inherent to observational database studies, such as injury severity, fracture morphology, and surgeon-specific decision-making. Only patients with complete data for all matching variables were included, and no imputation was performed.

Outcomes were identified using International Classification of Diseases, Tenth Revision, Clinical Modification (ICD-10-CM) and Current Procedural Terminology (CPT) codes. Reoperation-related outcomes were identified using Current Procedural Terminology (CPT) codes, whereas diagnoses-based outcomes, including infection, wound dehiscence, and other complications, were identified using International Classification of Diseases, Tenth Revision, Clinical Modification (ICD-10-CM) codes. Outcomes were defined using prespecified groupings of related ICD-10-CM or CPT codes rather than individual codes alone. Because outcomes were derived from administrative coding data, misclassification may occur due to variability in documentation practices and coding accuracy.

Primary outcomes included cumulative secondary surgeries at 1 and 2 years, defined as revision operations, irrigation and debridement (I & D), removal of hardware, amputations through the tibia or fibula, and other fracture-related procedures. Secondary outcomes were assessed at 90 days and included infection, wound dehiscence, a composite of infection or wound dehiscence, and deep peroneal nerve injury. Pseudoarthrosis and malunion/nonunion were also evaluated at 1 and 2 years in the respective cohorts. Outcomes with low event counts (≤10) were suppressed according to TriNetX privacy policies or pooled into composite endpoints when appropriate.

Risk ratios (RR) with 95% confidence intervals and absolute risk differences (ARD) were calculated, with p-values derived from Chi-square or Fisher's exact tests as appropriate. A two-sided p-value <0.05 was considered statistically significant. All codes used for querying, propensity-score matching, and outcome measurement are available in Supplementary Table 1.

## 3 Results

### 3.1 Cohort identification & matching

Prior to 1:1 propensity score matching of all operatively treated (open and closed) trimalleolar fractures, the TTCA cohort included 210 patients, whereas the ORIF cohort comprised 24,130 patients. Following matching, both cohorts were balanced at 210 patients each, with adequate covariate balance achieved (all standardized mean differences <0.10), except for a small residual imbalance in type 2 diabetes mellitus with circulatory complications (SMD = 0.1147) (Table 1).

**Table 1:** Cohort characteristics before and after propensity score matching among patients with operatively treated trimalleolar fractures (TTCA vs ORIF). TTCA: tibiotalocalcaneal arthrodesis; ORIF: open reduction and internal fixation; Std Diff: standardized mean difference; SD: standard deviation; BMI: body mass index.

Variable	Before Matching TTCA (n = 210)	Before Matching ORIF (n = 24,130)	Before Matching Std Diff	After Matching TTCA (n = 210)	After Matching ORIF (n = 210)	After Matching Std Diff
Age, years (mean ± SD)	62.6 ± 13.9	51.6 ± 18.0	0.6827	62.6 ± 13.9	63.8 ± 13.6	0.0873
Female, n (%)	128 (61.0%)	16,908 (70.1%)	0.1927	128 (61.0%)	130 (61.9%)	0.0196
Male, n (%)	82 (39.0%)	7220 (29.9%)	0.1929	82 (39.0%)	80 (38.1%)	0.0196
Type 2 diabetes mellitus, n (%)	116 (55.2%)	3813 (15.8%)	0.9044	116 (55.2%)	116 (55.2%)	<0.0001
Type 2 diabetes mellitus with neurologic complications, n (%)	69 (32.9%)	1159 (4.8%)	0.7688	69 (32.9%)	66 (31.4%)	0.0306
Type 2 diabetes mellitus with circulatory complications, n (%)	17 (8.1%)	363 (1.5%)	0.3121	17 (8.1%)	11 (5.2%)	0.1147
BMI 30–39, n (%)	56 (26.7%)	2875 (11.9%)	0.3806	56 (26.7%)	57 (27.1%)	0.0107
BMI ≥40, n (%)	35 (16.7%)	1461 (6.1%)	0.3392	35 (16.7%)	35 (16.7%)	<0.0001
Osteoporosis without pathologic fracture, n (%)	32 (15.2%)	1620 (6.7%)	0.2753	32 (15.2%)	29 (13.8%)	0.0406
Tobacco use, n (%)	13 (6.2%)	1183 (4.9%)	0.0563	13 (6.2%)	18 (8.6%)	0.0912
Osteoporosis with pathologic fracture, n (%)	10 (4.8%)	260 (1.1%)	0.2202	10 (4.8%)	10 (4.8%)	<0.0001
Peripheral vascular disease, n (%)	28 (13.3%)	791 (3.3%)	0.3706	28 (13.3%)	22 (10.5%)	0.0883
Dementia, n (%)	13 (6.2%)	321 (1.3%)	0.2576	13 (6.2%)	15 (7.1%)	0.0382

In the sensitivity analysis limited to closed trimalleolar fractures, the TTCA and ORIF cohorts initially included 148 and 23,576 patients, respectively. After propensity score matching, both cohorts comprised 147 patients, with adequate covariate balance achieved across all matching variables (SMDs <0.10) (Table 2).

**Table 2:** Cohort characteristics before and after propensity score matching among patients with operatively treated closed trimalleolar fractures (TTCA vs ORIF). TTCA: tibiotalocalcaneal arthrodesis; ORIF: open reduction and internal fixation; Std Diff: standardized mean difference; SD: standard deviation; BMI: body mass index.

Variable	Before Matching TTCA (n = 148)	Before Matching ORIF (n = 23,576)	Before Matching Std Diff	After Matching TTCA (n = 147)	After Matching ORIF (n = 147)	After Matching Std Diff
Age, years (mean ± SD)	64.1 ± 13.2	51.4 ± 17.9	0.8096	64.1 ± 13.2	64.3 ± 13.5	0.0184
Female, n (%)	99 (66.9%)	16,617 (70.5%)	0.0775	98 (66.7%)	97 (66.0%)	0.0144
Male, n (%)	49 (33.1%)	6957 (29.5%)	0.0777	49 (33.3%)	50 (34.0%)	0.0144
Type 2 diabetes mellitus, n (%)	90 (60.8%)	3748 (15.9%)	1.0414	89 (60.5%)	89 (60.5%)	<0.0001
Type 2 diabetes mellitus with neurologic complications, n (%)	56 (37.8%)	1133 (4.8%)	0.8813	55 (37.4%)	55 (37.4%)	<0.0001
Type 2 diabetes mellitus with circulatory complications, n (%)	16 (10.8%)	361 (1.5%)	0.3930	15 (10.2%)	11 (7.5%)	0.0959
BMI 30–39, n (%)	47 (31.8%)	2876 (12.2%)	0.4861	46 (31.3%)	48 (32.7%)	0.0292
BMI ≥40, n (%)	31 (20.9%)	1422 (6.0%)	0.4474	30 (20.4%)	30 (20.4%)	<0.0001
Osteoporosis without pathologic fracture, n (%)	24 (16.2%)	1596 (6.8%)	0.2995	23 (15.6%)	21 (14.3%)	0.0381
Tobacco use, n (%)	12 (8.1%)	1208 (5.1%)	0.1203	12 (8.2%)	11 (7.5%)	0.0253
Osteoporosis with pathologic fracture, n (%)	10 (6.8%)	246 (1.0%)	0.2984	10 (6.8%)	10 (6.8%)	<0.0001
Peripheral vascular disease, n (%)	28 (18.9%)	780 (3.3%)	0.5127	27 (18.4%)	26 (17.7%)	0.0177
Dementia, n (%)	11 (7.4%)	308 (1.3%)	0.3031	10 (6.8%)	10 (6.8%)	<0.0001

### 3.2 All trimalleolar fractures analysis

**Fig. 1:**
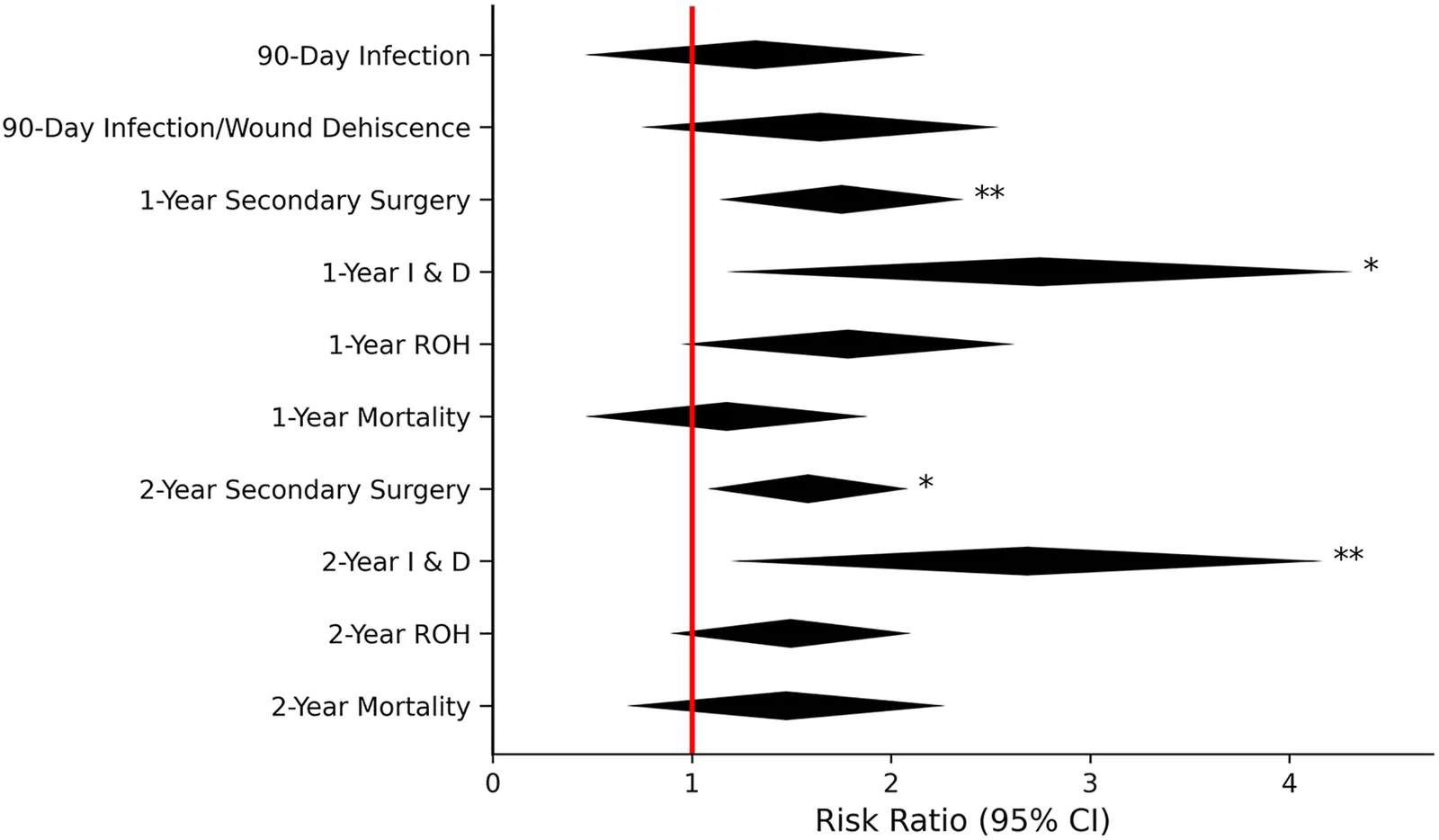
Postoperative outcomes following operative fixation (TTCA vs. ORIF) of trimalleolar fractures**.** TTCA, tibiotalocalcaneal arthrodesis; ORIF, open reduction internal fixation; CI, confidence interval, I & D, irrigation and debridement; ROH: removal of hardware. *p < 0.05, **p < 0.01.

Postoperative outcomes for patients undergoing TTCA versus ORIF for all (including both open and closed) trimalleolar fractures are summarized in Table 3 and Fig. 1.

**Table 3:** Postoperative outcomes following operative fixation (TTCA vs. ORIF) of both open and closed trimalleolar fractures**.** TTCA: tibiotalocalcaneal arthrodesis; ORIF: open reduction internal fixation; RR: risk ratio; CI: confidence interval, I & D: irrigation and debridement; ROH: removal of hardware.

Variable	TTCA Events (Risk %) (n = 210)	ORIF Events (Risk %) (n = 210)	Absolute Risk Difference (%)	RR (95% CI)	p-value
90-Day Infection	12 (5.7%)	12 (5.7%)	0.0	1.000 (0.460–2.175)	1.000
90-Day Infection or Wound Dehiscence	22 (10.5%)	16 (7.6%)	2.9	1.375 (0.743–2.543)	0.307
1-Year Secondary Surgery	59 (28.1%)	36 (17.1%)	11.0	1.639 (1.135–2.367)	**0.007**
1-Year I & D	27 (12.9%)	12 (5.7%)	7.2	2.250 (1.172–4.321)	**0.012**
1-Year ROH	33 (15.7%)	21 (10.0%)	5.7	1.571 (0.941–2.624)	0.080
1-Year Mortality	14 (6.7%)	15 (7.1%)	0.4	0.933 (0.462–1.885)	0.847
2-Year Secondary Surgery	66 (31.4%)	44 (21.0%)	10.4	1.500 (1.078–2.087)	**0.015**
2-Year I & D	29 (13.8%)	13 (6.2%)	7.6	2.231 (1.193–4.170)	**0.009**
2-Year ROH	41 (19.5%)	30 (14.3%)	5.2	1.367 (0.889–2.102)	0.152
2-Year Mortality	21 (10.0%)	17 (8.1%)	1.9	1.235 (0.671–2.274)	0.496

#### 3.2.1 90-Day outcomes

At 90 days, TTCA was associated with similar rates of infection (5.7% vs 5.7%) and the composite of infection and wound dehiscence (10.5% vs 7.6%) compared with ORIF; none of these differences reached statistical significance. Event counts for wound dehiscence and deep peroneal nerve injury were below the reporting threshold and could not be analyzed.

#### 3.2.2 1-Year outcomes

At 1 year, TTCA was associated with higher rates of secondary surgery compared with ORIF (28.1% vs 17.1%; ARD 11.0%; RR 1.639, 95% CI 1.135–2.367; p = 0.007) and increased risk of I & D (12.9% vs 5.7%; ARD 7.2%; RR 2.250, 95% CI 1.172–4.321; p = 0.012). Rates of hardware removal were higher with TTCA (15.7% vs 10.0%) but did not reach statistical significance (RR 1.571, 95% CI 0.941–2.624; p = 0.080). One-year mortality for TTCA patients was 14 of 210 patients (6.7%) compared to 15 of 210 (7.1%) for ORIF patients (p = 0.847). Event counts for malunion/nonunion, pseudoarthrosis, and leg amputation remained below the TriNetX reporting threshold (<11 events) to protect against patient deidentification, thereby precluding analysis of these outcomes.

#### 3.2.3 2-Year outcomes

At 2 years, TTCA continued to demonstrate higher rates of secondary surgery (31.4% vs 21.0%; RR 1.500, 95% CI 1.078–2.087; p = 0.015) and I & D (13.8% vs 6.2%; RR 2.231, 95% CI 1.193–4.170; p = 0.009). Hardware removal remained higher in TTCA (19.5% vs 14.3%) but was not statistically significant (RR 1.367, 95% CI 0.889–2.102; p = 0.152). Two-year mortality rate for TTCA patients was 21 of 210 patients (10.0%) compared to 17 of 210 (8.1%) for ORIF patients (p = 0.496). Counts for malunion/nonunion, pseudoarthrosis, and leg amputation remained below the TriNetX reporting threshold, precluding analysis of these outcomes.

### 3.3 Sensitivity analysis – closed fractures

**Fig. 2:**
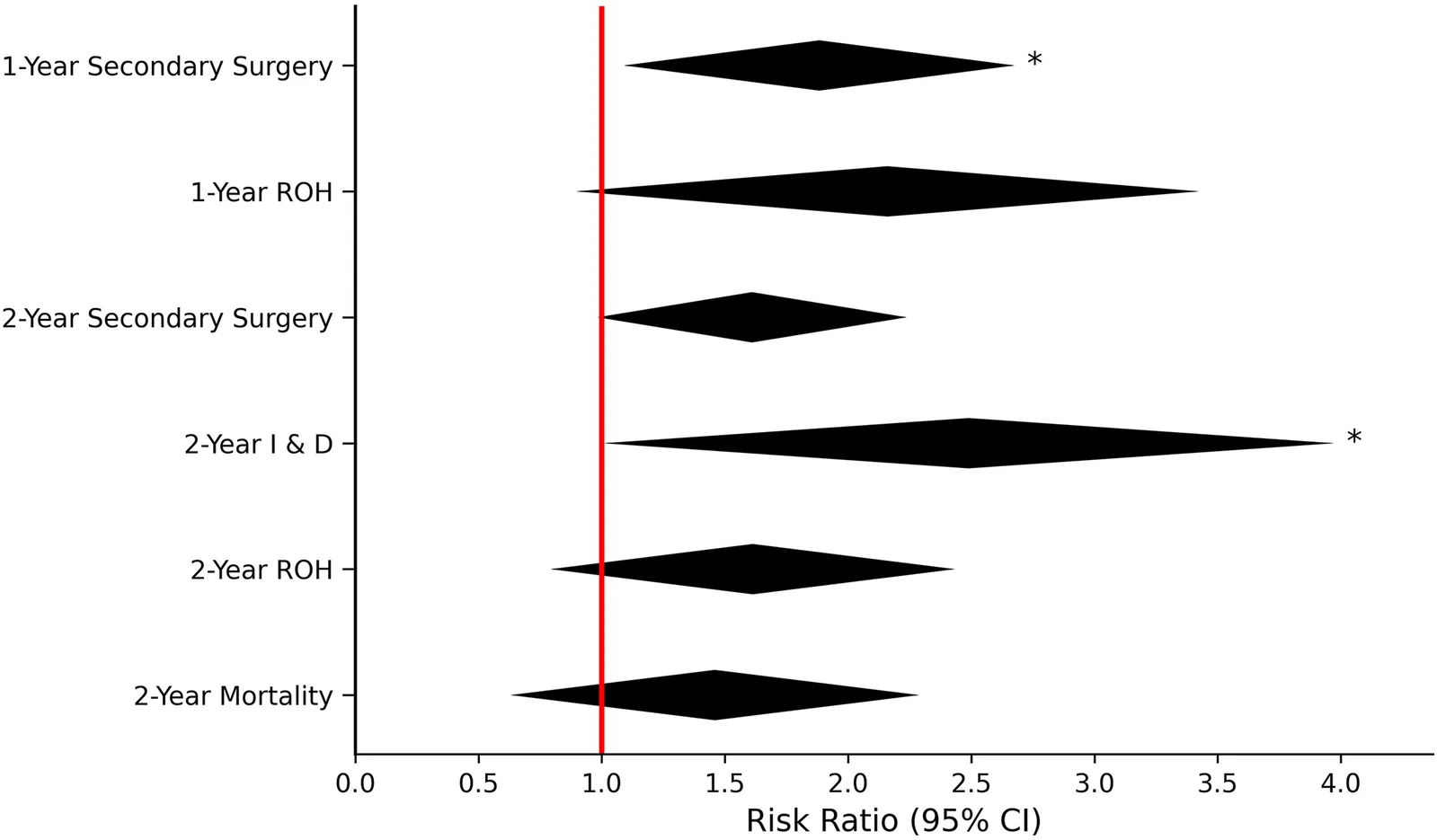
Postoperative outcomes following operative fixation (TTCA vs. ORIF) of closed trimalleolar fractures. TTCA, tibiotalocalcaneal arthrodesis; ORIF, open reduction internal fixation; CI, confidence interval, I & D, irrigation and debridement; ROH: removal of hardware. *p < 0.05.

Postoperative outcomes for patients undergoing TTCA versus ORIF for closed trimalleolar fractures only are summarized in Table 4 and Fig. 2.

**Table 4:** Postoperative outcomes following operative fixation (TTCA vs. ORIF) of only closed trimalleolar fractures**.** TTCA: tibiotalocalcaneal arthrodesis; ORIF: open reduction internal fixation; RR: risk ratio; CI: confidence interval, I & D: irrigation and debridement; ROH: removal of hardware.

Variable	TTCA Events (Risk %) (n = 147)	ORIF Events (Risk %) (n = 147)	Absolute Risk Difference (%)	RR (95% CI)	p-value
1-Year Secondary Surgery	41 (27.9%)	24 (16.3%)	11.6	1.708 (1.090–2.676)	**0.017**
1-Year ROH	21 (14.3%)	12 (8.2%)	6.1	1.750 (0.894–3.425)	0.096
2-Year Secondary Surgery	43 (29.3%)	29 (19.7%)	9.6	1.483 (0.982–2.238)	0.058
2-Year I & D	22 (15.0%)	11 (7.5%)	7.5	2.000 (1.006–3.974)	**0.042**
2-Year ROH	25 (17.0%)	18 (12.2%)	4.8	1.389 (0.792–2.434)	0.248
2-Year Mortality	18 (12.2%)	15 (10.2%)	2.0	1.200 (0.629–2.289)	0.579

#### 3.3.1 90-Day outcomes

In the sensitivity analysis restricted to closed fractures, 90-day complications were infrequent, with insufficient event counts for analysis of infection, wound dehiscence, the composite outcome of infection and wound dehiscence, and deep peroneal nerve injury.

#### 3.3.2 1-Year outcomes

At 1 year, TTCA was associated with higher rates of secondary surgery compared with ORIF (27.9% vs 16.3%; ARD 11.6%; RR 1.708, 95% CI 1.090–2.676; p = 0.017). Rates of hardware removal were higher with TTCA (14.3% vs 8.2%) but did not reach statistical significance (RR 1.750, 95% CI 0.894–3.425; p = 0.096). Event counts for I & D and mortality were too low to report for either cohort, precluding analysis of these outcomes.

#### 3.3.3 2-Year outcomes

At 2 years, TTCA demonstrated higher rates of I & D (15.0% vs 7.5%; RR 2.000, 95% CI 1.006–3.974; p = 0.042). Secondary surgery remained higher following TTCA (29.3% vs 19.7%; RR 1.483, 95% CI 0.982–2.238; p = 0.058) but did not reach statistical significance. Hardware removal was also higher following TTCA (17.0% vs 12.2%; RR 1.389, 95% CI 0.792–2.434; p = 0.248). Two-year mortality rate for TTCA patients was 18 of 147 patients (12.2%) compared to 15 of 147 (10.2%) for ORIF patients (p = 0.579).

## 4 Discussion

The principal finding of this study is that tibiotalocalcaneal arthrodesis (TTCA) was associated with significantly higher rates of secondary surgery and irrigation and debridement (I&D) at both 1 and 2 years postoperatively compared with open reduction and internal fixation (ORIF), despite similar early wound complication rates. These findings persisted in a sensitivity analysis restricted to closed fractures, suggesting a consistent association across cohorts with comparable baseline comorbidity profiles.

TTCA has been a standard option in the treatment of hindfoot arthritis and deformity for several decades and has more recently been applied to the management of unstable ankle fractures and pilon fractures. Its use has expanded particularly in two challenging populations: geriatric patients with fragility ankle fractures following low-energy mechanisms, and trauma patients with high-grade open injuries. Non-operative treatment of geriatric and high-risk (diabetic/neuropathic) fractures is associated with substantial morbidity, including pressure ulcers, loss of reduction requiring conversion to ORIF (19%),^15^ malunion (15%), progressive deformity, as well as increased 1-year mortality (9.2–21.5%) compared with operative management (6.7–9.1%), which has been shown to be protective.^16^^,^^17^ Operative treatment, however, is not without risk and carries complication rates including wound complications and infection (10%),^15^ fixation failure (1.7%),^15^ and secondary surgery, including arthroplasty, arthrodesis, hardware removal, and amputation.^17^ These competing risks underscore the complexity of treating ankle fractures in an increasingly elderly and medically frail population.^18^

A theoretical advantage of TTCA in this population is early mobilization due to a rigid construct with compression of both the tibiotalar and subtalar joints, less soft tissue disruption in a typically poor soft tissue envelope – particularly when joint preparation is omitted -, and maintenance of multiplanar alignment. This is reflected in the older age of patients treated with TTCA prior to matching in the present study (62.6 vs 51.6 years), suggesting preferential use in higher-risk patients. However, the benefit of early weightbearing may be less distinct, as postoperative protocols following ORIF remain variable and emerging evidence supports early mobilization with comparable safety and improved short-term outcomes.^19^, ^20^, ^21^ These data largely reflect lower-risk populations, as many studies exclude patients with higher BMI, complex fracture patterns, syndesmotic injury, or advanced age. Despite these proposed advantages, the present findings demonstrate increased midterm complications associated with TTCA.

At 90 days, the composite rate of wound complications and infection was higher in the TTCA cohort (10.5% vs 7.6%), although this difference did not reach statistical significance. Even modest increases in early wound complications may be clinically meaningful in this population, often necessitating antibiotics, immobilization, or hospital readmission. These treatments are not without increased care costs and side effects such as gastrointestinal upset, nosocomial infection, urinary tract infection, pneumonia, deep vein thrombosis, delirium, renal failure, and cardiac events – all of which can be detrimental to this already vulnerable population in the early post-operative period.

At 1 year, TTCA was associated with significantly higher rates of secondary surgery (28.1% vs 17.1%; RR 1.64, p = 0.007) and I&D (12.9% vs 5.7%; RR 2.25, p = 0.012). Secondary surgery in this cohort can include revision for malunion or nonunion, removal of hardware for broken or symptomatic implants, or infection in the form of amputation. The underlying etiology of increased reoperation rates following TTCA is likely multifactorial. One important limitation is the inability to stratify by surgical technique within the TriNetX database, particularly with respect to joint preparation. Prior work has suggested that omission of joint preparation may influence reoperation rates. A multicenter study by Kim et al. evaluated 149 patients undergoing TTCA for rotational ankle and pilon fractures, reporting that 24% underwent joint preparation, with improved rates of arthrodesis (94% vs 24%) but no significant difference in fracture union (81% vs 66%). They also observed a trend toward higher nonunion rates in patients undergoing joint preparation (19% vs 8%).^22^ Although not specific to rotational ankle fractures, these findings suggest that joint preparation may influence certain outcomes without clearly improving fracture healing, and this variability may contribute to the differences observed in the present study.^22^

The elevated rate of I&D in the TTCA cohort within the first postoperative year (12.9%) further highlights infection as a key driver of complications. A prior propensity-matched study of 462 patients undergoing TTCA for traumatic indications reported a 9% rate of infectious complications (wound dehiscence, cellulitis, or infectious symptoms) and a 22.2% all-cause revision rate, compared with 8.1% in patients undergoing TTCA for osteoarthritis.^23^ This finding is likely secondary to the soft tissue injury sustained in unstable ankle fractures leading to higher rates of secondary surgery and I&D, underscoring the importance of meticulous soft tissue management intra-operatively and edema management post-operatively when treating ankle fractures with primary TTCA.

Prior to propensity matching, there was a higher incidence of TTCA utilization among patients with BMI >30 (26.7% of TTCA patients with BMI 30–39 and 16.7% with BMI ≥40, compared to 11.9% and 6.1%, respectively, in the ORIF cohort), as well as in patients with osteoporosis without pathologic fracture (15.2% vs 6.7%). Additionally, 55.2% of patients treated with TTCA had type 2 diabetes mellitus, of whom 32.9% had associated neuropathy and 8.1% had circulatory complications. In contrast, only 15.8% of patients in the ORIF cohort had type 2 diabetes mellitus, with 4.8% demonstrating neuropathic involvement and 1.5% circulatory complications. These pre-matching differences highlight the preferential use of TTCA in a higher-risk patient population, particularly those with metabolic disease and end-organ complications, in whom the procedure is often selected with the intent of mitigating complications associated with traditional fixation strategies. A recent study reported an 18.5% overall complication rate in patients treated with primary TTCA for complicated diabetic ankle fractures, with 4 of 27 patients (14.8%) requiring secondary surgery for infectious or wound-related complications,^13^ which is comparable to the rates of secondary I&D observed within the first postoperative year in the present study.

Differences in surgeon practice patterns may also contribute to the observed increase in secondary surgery rates. Elective removal of hindfoot nails, particularly in cases without joint preparation, may increase reoperation rates in the TTCA cohort. Additionally, prior comparative studies have demonstrated higher complication rates with TTCA relative to ORIF. Stake et al. reported a 27% overall complication rate in the nail cohort compared with 18% in the ORIF cohort.^24^ Similar trends were observed in the present study, including in the sensitivity analysis restricted to closed fractures.

This study has several limitations. Given the use of a large multicenter database, patient identification, procedures, and outcomes were defined using administrative coding (ICD, CPT, and prescription data), which introduces the potential for misclassification and limits clinical granularity. Misclassification of procedures and outcomes may have led to under- or overestimation of event rates, potentially biasing observed associations toward or away from the null depending on coding accuracy. Important variables—including fracture severity, soft tissue condition, surgical technique (including joint preparation), and implant characteristics—could not be assessed. Although propensity score matching was performed, residual confounding likely persists, particularly related to unmeasured variables such as frailty, functional status, and indication for TTCA versus ORIF. This introduces the potential for selection bias, as patients undergoing TTCA may have been inherently at higher risk for complications. Additionally, patient-reported outcomes, ambulatory status, and detailed indications for secondary surgery (e.g., infection, nonunion, elective hardware removal) were not available. Variability in operative technique across institutions—including differences in joint preparation, fixation constructs, and postoperative protocols—represents an additional source of heterogeneity. Finally, longer-term follow-up would strengthen this analysis by capturing late complications such as post-traumatic arthrosis and progressive deformity.

## 5 Conclusion

While previously considered a less invasive limb salvage option for ankle fractures in vulnerable patient populations, primary TTCA may be associated with a higher incidence of secondary surgery—particularly irrigation and debridement—at both one and two years postoperatively compared with ORIF, although these findings may be influenced by residual confounding despite propensity matching. Future prospective studies are needed to better define the optimal management of fractures in this clinically challenging population.

## Patient consent

The data used in this retrospective cohort study was collected from the TriNetX Research Network, which provided access to electronic medical records (diagnoses, procedures, medications, laboratory values, genomic information) from over 150 million patients from 102 healthcare organizations. TriNetX is compliant with the Health Insurance Portability and Accountability Act (HIPAA), certified to the ISO 27001:2013 standard, and maintains an Information Security Management System (ISMS) to protect healthcare data. All patient-level data are de-identified according to HIPAA standards. Because only de-identified records were used, the study was exempt from Institutional Review Board approval.

## Credit author statement

**Ronak J. Mahatme, MD:** Conceptualization, Methodology, Supervision, Project administration, Visualization, Writing – review & editing; **Meredith Murphy, MD:** Conceptualization, Methodology, Supervision, Project administration, Writing – review & editing; **Shawn A. Moore, BS:** Software, Investigation, Formal analysis, Visualization, Writing – review & editing; **Anish Gangavaram, BS:** Software, Investigation, Formal analysis, Visualization, Writing – original draft, Writing – review & editing; **Caroline Szabo, BS:** Writing – original draft, Writing – review & editing**; Abraar Huq, BS:** Investigation, Writing – original draft, Writing – review & editing; **Richard Laughlin, MD:** Conceptualization, Methodology, Supervision, Project administration, Writing – review & editing.

## Ethical statement

This study provides a truthful and accurate representation of the work conducted, with data reported faithfully and methods described in sufficient detail to allow reproducibility. The manuscript is original, and any use of others’ work has been appropriately cited, quoted, and permissions obtained where required. This work has not been previously published, in whole or in part, and is not currently under consideration by another journal. All authors have approved the manuscript and its submission, and the appropriate institutional authorities have approved the work where required. If accepted, the manuscript will not be published elsewhere in the same form, in any language, without the written consent of the copyright holder.

## Funding

This research did not receive any specific grant from funding agencies in the public, commercial, or not-for-profit sectors.

## Declaration of competing interest

The authors declare that they have no known competing financial interests or personal relationships that could have appeared to influence the work reported in this paper.

## References

[1] Pina G., Fonseca F., Vaz A., Carvalho A., Borralho N. (2021). Unstable malleolar ankle fractures: evaluation of prognostic factors and sports return. Arch Orthop Trauma Surg.

[2] Stead T.S., Pomerantz L.H., Ganti L. (2021). Acute management of trimalleolar fracture. Cureus.

[3] Balziano S., Baran I., Prat D. (2023). Hindfoot nailing without joint preparation for ankle fractures in extremely elderly patients: comparison of clinical and patient-reported outcomes with standard ORIF. Foot Ankle Surg.

[4] Testa G., Ganci M., Amico M. (2019). Negative prognostic factors in surgical treatment for trimalleolar fractures. Eur J Orthop Surg Traumatol.

[5] Gougoulias N, Oshba H, Dimitroulias A, Sakellariou A, Wee A. Ankle fractures in diabetic patients. Published online August 3, 2020. Accessed March 26, 2026. https://eor.bioscientifica.com/view/journals/eor/5/8/2058-5241.5.200025.xml. 10.1302/2058-5241.5.200025 PMC7484713 32953131

[6] Lopez-Capdevila L., Ruh J.M.R., Vidal J.F. (2019). Diabetic ankle fracture complications: a meta-analysis. Foot Ankle Orthopaedics.

[7] Jones K.B., Maiers-Yelden K.A., Marsh J.L., Zimmerman M.B., Estin M., Saltzman C.L. (2005). Ankle fractures in patients with diabetes mellitus. J Bone Joint Surg Br.

[8] Kirchner G.J., Kim A.H., Martinazzi B.J., Sudah S.Y., Lieber A.M., Aynardi M.C. (2023). Factors associated with amputation following ankle fracture surgery. J Foot Ankle Surg.

[9] Georgiannos D., Lampridis V., Bisbinas I. (2017). Fragility fractures of the ankle in the elderly: open reduction and internal fixation versus tibio-talo-calcaneal nailing: short-term results of a prospective randomized-controlled study. Injury.

[10] Phillips M., Bullock G., Scott A. (2022). Use of the straight retrograde intramedullary nail for tibiotalocalcaneal arthrodesis: a single-institution, single-surgeon, single-implant retrospective series. Foot Ankle Orthopaedics.

[11] Shah A.B., Jones C., Elattar O., Naranje S.M. (2017). Tibiotalocalcaneal arthrodesis with intramedullary fibular strut graft with adjuvant hardware fixation. J Foot Ankle Surg.

[12] Washburn F., Ahankoob N., Bonavida V., Fang W., Pyle C. (2024). Arthroscopic-assisted tibiotalocalcaneal arthrodesis using a hindfoot nail for treatment of ankle fractures in medically complex patients: a technique guide and retrospective case series. Tech Foot Ankle Surg.

[13] Ebaugh M.P., Umbel B., Goss D., Taylor B.C. (2019). Outcomes of primary tibiotalocalcaneal nailing for complicated diabetic ankle fractures. Foot Ankle Int.

[14] Dawar A., Chundi G., Fuller Z. (2025). Tibiotalocalcaneal arthrodesis vs open reduction internal fixation for trimalleolar ankle fractures in high-risk patients: a national database analysis. Foot Ankle Orthopaedics.

[15] Willett K., Keene D.J., Mistry D. (2016). Close contact casting vs surgery for initial treatment of unstable ankle fractures in older adults: a randomized clinical trial. JAMA.

[16] Bariteau J.T., Hsu R.Y., Mor V., Lee Y., DiGiovanni C.W., Hayda R. (2015). Operative versus nonoperative treatment of geriatric ankle fractures: a medicare part A claims database analysis. Foot Ankle Int.

[17] Koval K.J., Zhou W., Sparks M.J., Cantu R.V., Hecht P., Lurie J. (2007). Complications after ankle fracture in elderly patients. Foot Ankle Int.

[18] Koval K.J., Lurie J., Zhou W. (2005). Ankle fractures in the elderly: what you get depends on where you live and who you see. J Orthop Trauma.

[19] Chen B., Ye Z., Wu J., Wang G., Yu T. (2024). The effect of early weight-bearing and later weight-bearing rehabilitation interventions on outcomes after ankle fracture surgery: a systematic review and meta-analysis of randomised controlled trials. J Foot Ankle Res.

[20] Egu C., Akil H., Hakim R.A. (2026). Early vs late weight bearing after ankle fracture fixation: a meta-analysis of randomized controlled trials. Foot Ankle Int.

[21] Wang C., Li C. (2025). Early weight-bearing after ankle fracture surgery: a systematic review and meta-analysis of functional outcomes and safety. J Orthop Surg Res.

[22] Kim E., Tornetta P., Carlson J.B. (2026). Factors affecting outcomes of hindfoot fusion nails for acute injury: a multicenter study. J Orthop Trauma.

[23] Dawar A., Chundi G., Ahn D.B. (2025). Risk factors and complications in tibiotalocalcaneal (TTC) arthrodesis: a nationwide database comparison between traumatic ankle fracture and osteoarthritis. Foot Ankle Orthop.

[24] Stake I.K., Ræder B.W., Gregersen M.G. (2023). Higher complication rate after nail compared with plate fixation of ankle fractures in patients aged 60 years or older: a prospective, randomized controlled trial. Bone Jt J.

