# Supplementary material for: Tibiotalocalcaneal arthrodesis versus open reduction and internal fixation in treatment of trimalleolar ankle fractures: A propensity-matched TriNetX database study
Source: J Clin Orthop Trauma. 2026 Jun 3;79:103511. doi: 10.1016/j.jcot.2026.103511 (PMC13263664; doi:10.1016/j.jcot.2026.103511)
Supplement: Supplementary file 1 [file mmc1.docx]

| **Supplementary Table 1:** Current Procedural Terminology (CPT) and International Classification of Diseases, Tenth Revision, Clinical Modification (ICD-10-CM) Used for Query. | |
| --- | --- |
| **Diagnosis** | **Codes** |
| Trimalleolar Fracture | S82.85 |
| Closed Trimalleolar Fracture | S82.851A, S82.852A, S82.853A, S82.854A, S82.855A, S82.856A |
| Ankle open reduction internal fixation | 0SHF04Z, 0SHG04Z, 1005272 |
| Ankle Fusion | 0SGF03Z, 0SGF04Z, 0SGF07Z, 0SGF0KZ, 0SGF33Z, 0SGF34Z, 0SGF37Z, 0SGF3JZ, 0SGF3KZ, 0SGF43Z, 0SGF44Z, 0SGF47Z, 0SGF4JZ, 0SGF4KZ, 0SGG03Z, 0SGG04Z, 0SGG07Z, 0SGG0JZ, 0SGG0KZ, 0SGG33Z, 0SGG34Z, 0SGG37Z, 0SGG3JZ, 0SGG3KZ, 0SGG43Z, 0SGG44Z, 0SGG47Z, 0SGG4JZ, 0SGG4KZ |
| Tarsal Fusion | 0SGJ03Z, 0SGJ04Z, 0SGJ07Z, 0SGJ0JZ, 0SGJ0KZ, 0SGJ33Z, 0SGJ34Z, 0SGJ37Z, 0SGJ3JZ, 0SGJ3KZ, 0SGJ43Z, 0SGJ44Z, 0SGJ47Z, 0SGJ4JZ, 0SGJ4KZ, 0SGH03Z, 0SGH04Z, 0SGH07Z, 0SGH0JZ, 0SGH0KZ, 0SGH33Z, 0SGH34Z, 0SGH37Z, 0SGH3JZ, OSGH3KZ, 0SGH43Z, 0SGH44Z, 0SGH47Z, 0SGH4JZ, 0SGH4KZ |
| Propensity Score Inputs | Age at Index, Sex, Z68.3, Z68.4, Z72.0, E11, M80-81, E11.4, I73, E11.5, F03 |
| Infection | T81.4 |
| Wound Dehiscence | T81.31, T81.32 |
| Peroneal Nerve Injury | S94.20XA, S94.21XA, S94.22XA |
| Malunion/Nonunion | S82.851P, S82.851Q, S82.851R, S82.851K, S82.851M, S82.851N, S82.852P, S82.852Q, S82.852R, S82.852K, S82.852M, S82.852N, S82.853P, S82.853Q, S82.853R, S82.853K, S82.853M, S82.853N, S82.854P, S82.854Q, S82.854R, S82.854K, S82.854M, S82.854N, S82.855P, S82.855Q, S82.855R, S82.855K, S82.855M, S82.855N, S82.856P, S82.856Q, S82.856R, S82.856K, S82.856M, S82.856N |
| Leg Amputation | 1014590 |
| Secondary Surgery | 1003735, 1005161, 1005233, 1014588, 1005272, 1005290, 27766, 27769, 27792, 27814, 27829, 27870, 1014590, 27704, 27726, 1005278, 1014596, 1014598, 28406, 28436, 28445, 28555, 28576, 28585, 28120, 28320, 28725, 1003173, 1020523, 1020524, 1020525 |
| Irrigation & Debridement | 1005161, 1003173, 1020523, 1020524, 1020525 |
| Removal of Hardware | 27704, 1003735 |
| Pseudoarthrosis | M96.0 |
| Mortality | Deceased |
